# Supplementary material for: MetaRibo-Seq measures translation in microbiomes
Source: Nat Commun. 2020 Jun 29;11:3268. doi: 10.1038/s41467-020-17081-z (PMC7324362; doi:10.1038/s41467-020-17081-z)
Supplement: Supplementary file 10 — Supplementary Data 7 [file 41467_2020_17081_MOESM10_ESM.zip › File2/Confidence_VeryHigh_Taxonomy/319022_out.krona.html]

Javascript must be enabled to view this page.

members
magnitude
magnitudeUnassigned
count
unassigned
taxon
rank

319022\_out

6

2
superkingdom
6

976
6
phylum

200643
class
6

171549
order
6

family
6
815

816
6
genus

1946088
species

SRS893327\_contig\_number\_5851
1

2292944
species

SRS893341\_contig\_number\_1515SRS893373\_contig\_number\_27585SRS971275\_contig\_number\_15609
3

2292941

SRS893369\_contig\_number\_2144SRS893383\_contig\_number\_8575
2
species
